# The Suite for the Assessment of Low-Level cues on Orientation (SALLO): The psychophysics of spatial orientation in virtual reality

**DOI:** 10.3758/s13428-023-02265-4

**Published:** 2023-11-06

**Authors:** Davide Esposito, Alice Bollini, Monica Gori

**Affiliations:** 1 U-VIP: Unit for Visually Impaired People, Center of Human Technology, Italian Institute of Technology, 16152 Genoa, Italy; 2 RAISE ecosystem, Genova, Italy

**Keywords:** Virtual reality, Psychophysics, Spatial orientation, Cue combination, Space representations

## Abstract

Spatial orientation is a complex ability that emerges from the interaction of several systems in a way that is still unclear. One of the reasons limiting the research on the topic is the lack of methodologies aimed at studying multimodal psychophysics in an ecological manner and with affordable settings. Virtual reality can provide a workaround to this impasse by using virtual stimuli rather than real ones. However, the available virtual reality development platforms are not meant for psychophysical testing; therefore, using them as such can be very difficult for newcomers, especially the ones new to coding. For this reason, we developed SALLO, the Suite for the Assessment of Low-Level cues on Orientation, which is a suite of utilities that simplifies assessing the psychophysics of multimodal spatial orientation in virtual reality. The tools in it cover all the fundamental steps to design a psychophysical experiment. Plus, dedicated tracks guide the users in extending the suite components to simplify developing new experiments. An experimental use-case used SALLO and virtual reality to show that the head posture affects both the egocentric and the allocentric mental representations of spatial orientation. Such a use-case demonstrated how SALLO and virtual reality can be used to accelerate hypothesis testing concerning the psychophysics of spatial orientation and, more broadly, how the community of researchers in the field may benefit from such a tool to carry out their investigations.

## Introduction

**Fig. 1:**
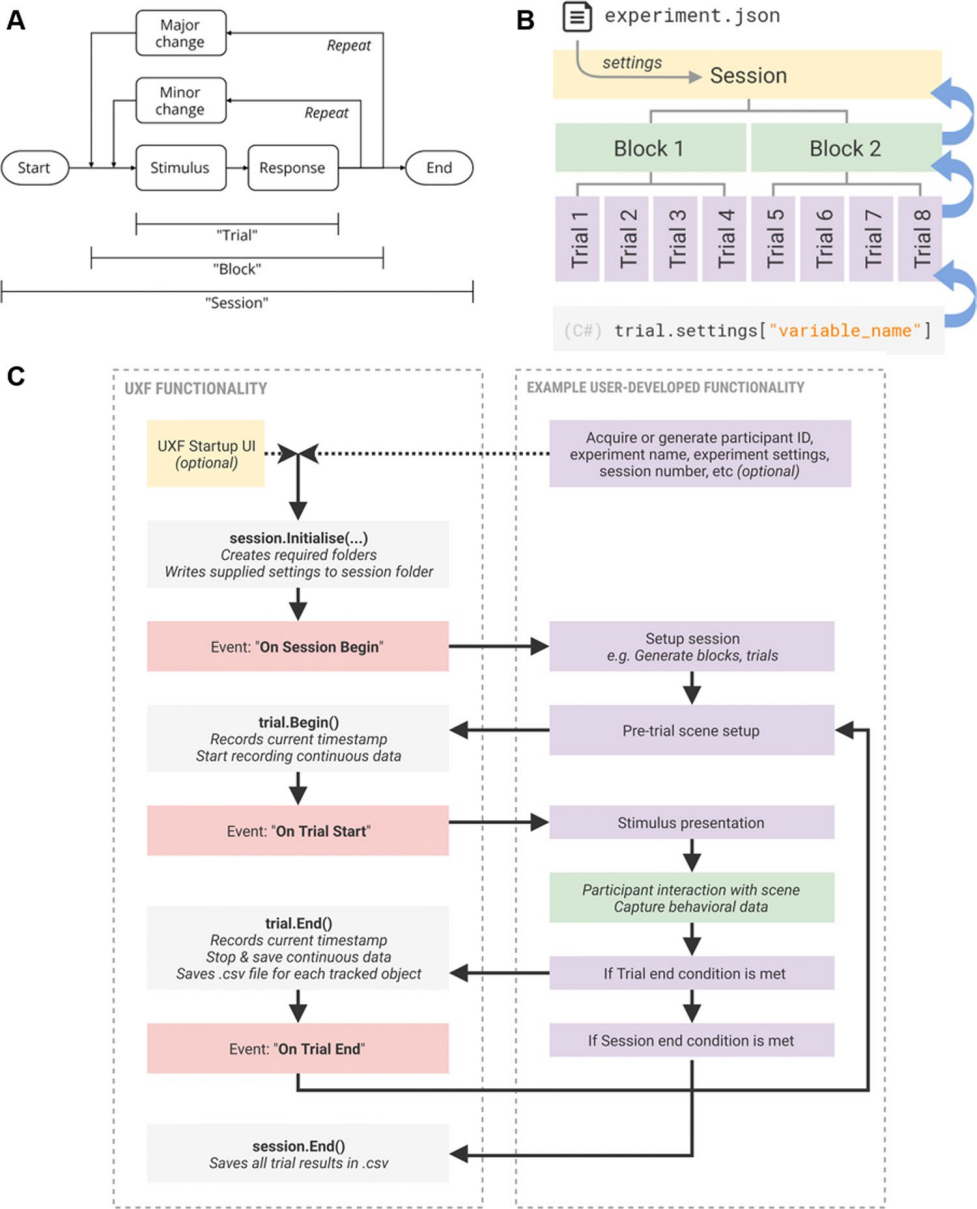
Major features of the Unity Experiment Framework (UXF). Block diagram describing one generic experimental procedure (**A**). Implementation of such procedure in the UXF (**B**). Flowchart for programming an experiment with the UXF. Reproduced from Brookes et al., 2020, under the Creative Commons 4.0 license

**Fig. 2:**
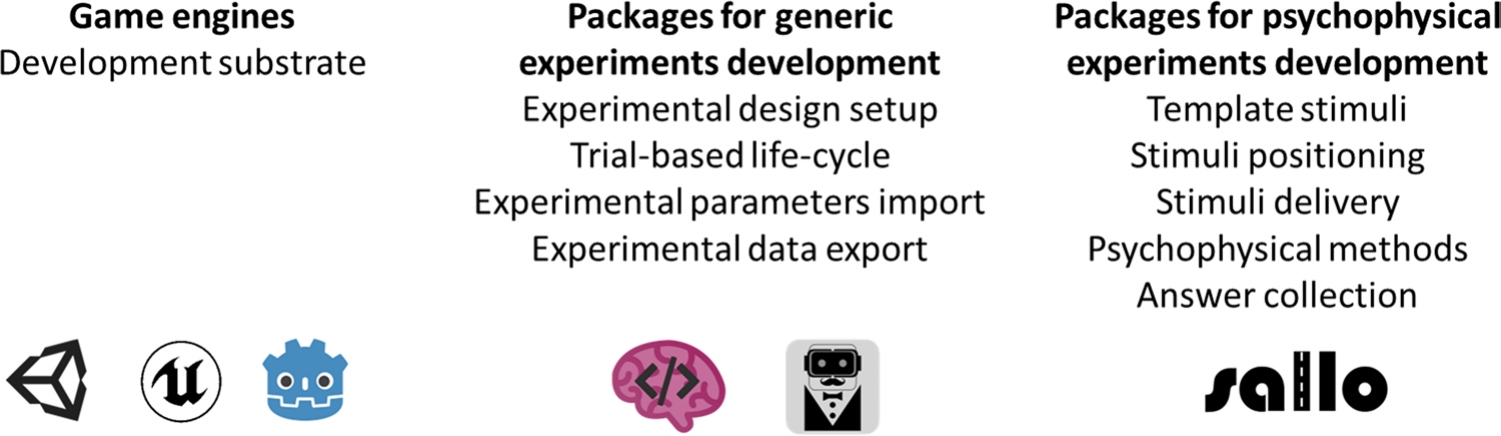
Comparison of SALLO’s added value compared to the existing tools for VR development. SALLO is a suite of tools tailored to the design of psychophysical experiments. As such, it does not contain tools for the design and execution of generic behavioral experiments in VR, but it relies the on existing ones, which in turn rely on the dedicated game engine they have been developed with

The ability to understand accurately and precisely the orientation of the surrounding items with respect to oneself and the other items in space is fundamental. As an example, a monkey escaping from a tiger, in order to maximize its survival chances, needs to understand the direction from which the tiger is arriving and identify the tree that is closer to itself and, at the same time, farther from the predator. This fundamental ability is, computationally speaking, quite intensive. As a matter of fact, it remains controversial how the sense of spatial orientation depends on the multiple low-level, sensori-motor cues that compose it (Berthoz & Viaud-Delmon, 1999; Epstein et al., 2017; Grieves & Jeffery, 2017). One of the obstacles to comprehending such links is the lack of methodologies to investigate complex percepts emerging from the interaction of multiple sensory and motor cues. Indeed, in psychophysics, the branch of psychology that aims to map physical stimuli to percepts (Gescheider, 2013), the effect under investigation is typically isolated to limit confounding factors. In a typical psychophysical experiment, participants must keep a specific body position, and their movements are often constrained. The stimuli used are simple and delivered using precise and accurate devices that give experimenters control over the physical dimension under investigation (Kingdom & Prins, 2016). This approach is powerful because it clearly outlines a mathematical relationship between stimulus and percept; indeed, it was born to map the perception of simple features, such as light intensity, mechanical pressure, or sound frequencies (Stevens, 1960). However, the experimental settings used in psychophysics are oversimplified compared to real-life conditions (De Gelder & Bertelson, 2003): as the percepts under investigation depart from the primary sensations, the stimulus-perception maps found in the lab may differ from those of everyday life’s dynamic and multisensory world, that is, the control over the stimulus properties precedes the results’ ecological validity (Holleman et al., 2020; Loomis et al., 1999). To overcome the limitations of classical, unimodal psychophysical paradigms, researchers in the field of spatial orientation have been proposing experimental paradigms based on more and more complex technological solutions. Some examples of experimental settings and devices used in this research domain are roto-translational chairs (Butler et al., 2010; Zanchi et al., 2022) and treadmills (Frissen et al., 2011), optical motion tracking systems (Kolarik et al., 2016), anechoic chambers (Zahorik et al., 1995), wall-sized speaker arrays (Populin, 2008), robotic manipulanda (Volpe et al., 2009). For example, Barnett-Cowan and colleagues developed a 3D motion simulator consisting of a seat mounted to the flange of a modified KUKA anthropomorphic robot arm to study the contribution of visual and vestibulo-kinaesthetic cues to the perception of self-motion along different axes (Barnett-Cowan et al., 2012). Whereas these complex tools provide researchers with precise control over multiple cues and return accurate measures, they are expensive and bulky, and some require dedicated rooms; as a result, only a few institutions can afford them. Virtual reality (VR) offers an alternative route to increase ecological validity without losing control over the stimulation delivered: it uses known psychophysical laws to simulate the presence of items in space. For example, to acoustically simulate the presence of a virtual object at a given angle, a VR application would deliver the sound via headphones, with specific time and intensity differences between the two channels: the same interaural time (ITD) and intensity, or level, differences (ILD) that the physical object placed at such angle would have elicited (Middlebrooks & Green, 1991). This way, the complexity of controlling the physical stimuli disappears, and the cost decreases. VR has been employed in behavioral experiments for the last two decades (Cogné et al., 2017; Loomis et al., 1999), and because of its versatility, it has been recognized as a valuable tool to study the sensori-motor system at potentially any processing level: from early-stage sensory processing (Cogné et al., 2017; Parseihian & Katz, 2012; Zanchi et al., 2022) to sensori-motor interactions (Esposito et al., 2021a, b, 2023), up until high-level representations such as memory and emotions (Cogné et al., 2017; Rus-Calafell et al., 2018). However, so far, VR has been used scarcely to extend the range of possible psychophysics-based experimental design; it has been used much more to simulate realistic scenarios and study high-level cognitive abilities such as spatial memory and spatial navigation strategies: please refer to Cogné et al., 2017, and Montana et al., 2019, for some recent reviews. As these reviews reported, the virtual stimuli used commonly are virtual environments such as mazes, streets, or rooms, which the user can freely navigate. Although more straightforward and controlled than natural environments, these virtual environments are still rich topographically and geometrically. Therefore, it is difficult to assess the contribution of single cues to the spatial ability under investigation, as a psychophysical assessment would require. One reason why VR has not been broadly adopted to study the psychophysics of spatial orientation may be that the graphics engines used to develop VR applications, such as Unity (Unity Technologies, 2019e) and Unreal (Epic Games, 2022), are not built to develop scientific experiments, but rather to be general-purpose tools (Unity Technologies, 2019h). This feature makes the development of scientific experiments with stock graphic engines somewhat inefficient (de la Rosa & Breidt, 2018). Some plugins have been developed to simplify designing experiments with graphic engines. One example is the "Unity Experiment Framework" (UXF) (Brookes et al., 2020), a Unity plugin that modifies Unity's native life cycle to make it an iteration of blocks and trials (Fig. 1) and provides other handy tools for scientific investigation, such as tools to track items and save data. Another example is the "BiomotionLab Toolkit for Unity Experiments" (bmlTUX) (Bebko & Troje, 2020), which provides a simple graphical user interface to design experiments in terms of variable entry, trial order, counterbalancing, randomization, and blocking. Such plugins try to reshape Unity to simplify the development of generic behavioral experiments, but they do not focus on psychophysics, let alone psychophysics of spatial orientation. To date, no packages provide tools focused on psychophysics (e.g., template stimuli, tasks, psychophysical methods) and on the study of spatial orientation from a low-level perspective. For this reason, we developed a Unity package tailored to the psychophysical assessments of sensori-motor cues over spatial orientation, called "Suite for the Assessment of Low-Level cues on Orientation" (SALLO). SALLO is a suite of utilities that gives experimenters control over participant positioning, audio-visual stimuli selection, stimuli delivery, and response methods (forced choices, pointing, and so on). It aims at simplifying psychophysical testing and results' replicability by employing VR. Figure 2 describes the added value of SALLO with respect to the existing tools for behavioral testing in VR. In the following, the methodological aspects of the suite will be discussed, and a validation experiment will be presented to demonstrate the utility and versatility of SALLO.

## Methodology

SALLO is a suite of tools that simplifies designing psychophysical assessments about spatial orientation in the virtual space of Unity. It was developed following the guidelines Kingdom and Prins drew to design a psychophysical experiment (Kingdom & Prins, 2016). In their book, the authors suggest that a psychophysical experiment comprises five separate elements: stimulus, task, method, analysis, and measure. Taking inspiration from Kingdom and Prins' guidelines, the tools in SALLO focus independently on the stimulus, the task, and the psychophysical method. Moreover, since SALLO focuses specifically on spatial orientation, it also includes tools for the spatial positioning of stimuli and participants. Finally, since SALLO focuses on the psychophysical experiment execution rather than the performance evaluation, it does not contain tools for analysis or measurement.

### The SALLO back-end

SALLO contains two types of tools: Unity Components (Unity—Manual: Introduction to Components, 2019b) and GameObject prefabs (Unity—Manual: Prefabs, 2019c). In Unity, GameObjects are the basic entities populating the virtual space (Unity—Manual: GameObjects, 2019a). They must have a position and orientation in the virtual space and can contain other GameObjects, thus working as local reference frames. A GameObject that contains other GameObjects is called "Parent" of the latter; in turn, the contained GameObjects are called "Children" of the former. Components are the entities that add functionality to the GameObjects (Unity—Manual: Introduction to Components, 2019b). Some examples of Components are rendering meshes, materials, audio players, or even custom C# classes. GameObject prefabs are preformatted GameObjects, each with their pool of Components and children GameObjects (Unity—Manual: Prefabs, 2019c). They are stored in dedicated files and can be instantiated in the virtual space whenever needed. The following sections will introduce the tools in SALLO, with sub-sections dedicated to each element of the psychophysical experiment.

#### Stimulus

**Fig. 3:**
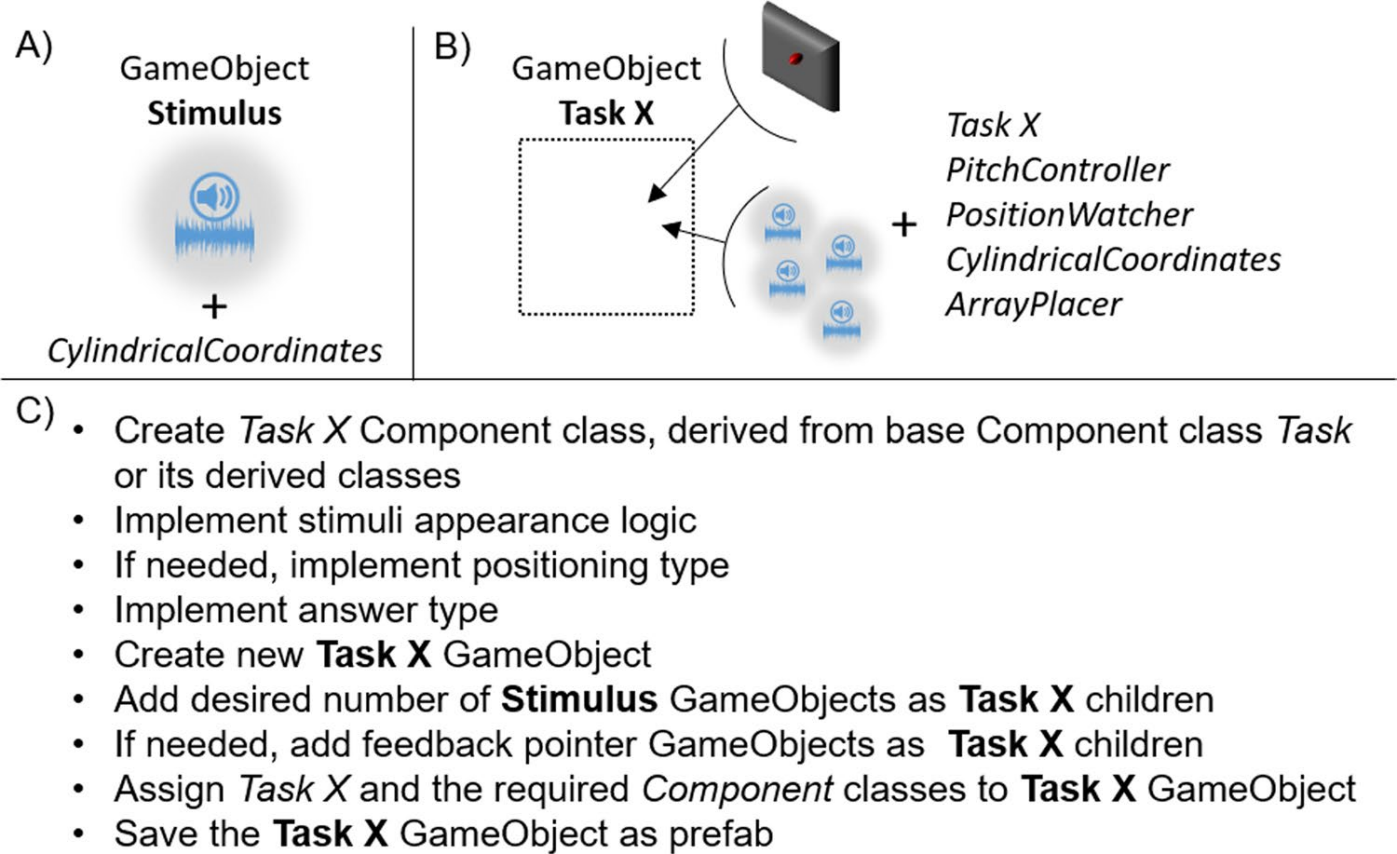
In panels A and B, schematic description of the "Stimulus" (**A**) and "Task" (**B**) GameObjects' appearance and components. In panel C, a step-by-step procedure to develop a custom task in SALLO

Although Unity offers all the tools to create any stimulus, they are not developed for psychophysics; therefore, they can be hard to use for researchers that are novice to Unity. Let’s take as examples two very common stimuli employed in psychophysics: a Gaussian blob and a stream of white noise. One way to create a Gaussian blob in Unity’s 3D environment from scratch is to: create a GameObject hierarchy with an empty GameObject as root, a sphere GameObject and a quad GameObject as children; set the sphere’s “material” Component properties (color, light emission, light reflection, texture, etc.) according to one’s needs; place the quad between the sphere and the observer’s point of view, change the quad’s shader with a custom one that implements the gaussian blur via code. Instead, to create a stream of white noise from scratch, one needs to: create a GameObject with an “AudioSource” Component; create a custom Component that fills the audio buffer with random numbers every time the system requests access to it. Self-implementing both these examples requires a good understanding of Unity’s audio-visual rendering workflow and a basic understanding of object-oriented programming. SALLO aims to reduce such minimum skills requirements by providing a sample audio-visual stimulus with desirable features to study audio-visual cross-modal effects in space. In fact, the basic stimulus that SALLO provides is a GameObject rendered as a light grey, blurred sphere emitting spatialized white noise generated in real-time (Fig. 3A). The stimulus dimension is arbitrarily set at 1 unity unit, the basic measurement unit for distance in the virtual space. This value is arbitrary because the actual stimulus dimension depends on the distance from the observer's virtual point of view, given the visual field size. The visual stimulus is blurred through a virtual surface placed between the stimulus and the observer, whose material was rendered with a custom shader (Unity Technologies, 2019f) that does not support single-pass stereo rendering (Unity Technologies, 2019g); therefore, multi-pass stereo rendering must be used (Unity Technologies, 2019g). The acoustic stimulus can be spatialized with any audio spatializer plugin. Two examples are the "Resonance Audio" plugin (Google, 2018) and the Unity wrapper for the "3D Tune-In" Toolkit (Cuevas-Rodríguez et al., 2019), an open-source library for real-time binaural spatialization.

#### Task

The next challenge in the design of a psychophysical experiment is deciding what task to use. To the best of our knowledge, no packages provide tools to simplify or templates and guidelines to standardize the development of a psychophysical task in Unity. SALLO filled the gap by providing those tools. Their design revolved around the argument that most psychophysical tasks for assessing spatial orientation share a common structure. We propose three features common to any generic psychophysical task for spatial orientation assessment: (i) the task delivers a set of stimuli (one or more) with a specific spatio-temporal structure; (ii) the task requires a trigger for the stimuli delivery (e.g., the previous trial's end or the participant reaching a specific orientation); (iii) the task requires an answer from the participant (e.g., head-pointing or 2AFC). Following these points, we defined a C# abstract class (i.e., a class that cannot be used per se) called "Task", from which any other class must inherit to implement specific behaviors. In the subsequent passages, the name "*Task X*" will identify a generic class derived from the "Task" base class. The "Task" class implements the methods for perceptual channel selection and forces every “*Task X*” class to implement the three common points mentioned above. Then, each "*Task X*" class will implement its specific behavior.

The "*Task X*" class defines the task logic, but a body is necessary as well to exist in the virtual environment. Therefore, SALLO requires the creation of a "Task X" GameObject prefab for each "*Task X*" class, with the corresponding "*Task X*" class assigned as a Component (Fig. 3B). SALLO already includes GameObject prefabs for some psychophysical tasks: localization (Zimmermann, 2021), repositioning (Roren et al., 2009), left–right discrimination (Lewald et al., 2000) and space bisection (Gori et al., 2014). We designed the SALLO tools presented in this subsection with the aim of standardizing the development of psychophysical tasks. Indeed, following the scheme summarized in Fig. 3C, experimenters can create their custom tasks and eventually share them with the community for reuse.

#### Psychophysical method

The third aspect SALLO handles is the psychophysical method. In the last century and a half, many methods have been developed, each with peculiar pros, cons, and best use cases (Kingdom & Prins, 2016). Typically, those methods are divided into non-adaptive and adaptive (Aleci, 2021). As the name suggests, non-adaptive methods choose the value for the feature of interest from a predefined pool unaffected by the previous participant's answers. Adaptive methods, instead, select the value for the feature of interest according to the previous participant's responses. In principle, adaptive methods require fewer trials than non-adaptive methods but make more assumptions about the percept's psychometric properties (Aleci, 2021). SALLO includes one method per category: the method of constant stimuli for what concerns non-adaptive procedures (Kingdom & Prins, 2016) and the QUEST method for what concerns adaptive procedures (Watson & Pelli, 1983). The method of constant stimuli was chosen because it is the most accurate non-adaptive method (Gescheider, 2013). The QUEST method was chosen because it is efficient (Watson & Fitzhugh, 1990). SALLO implements the desired psychophysical methods as classes derived from the base "*PsyMethod*" abstract C# class. It contains the list of desired values to test, the list of repetitions for each value, and a method to extract a randomized sequence of trials based on the desired values and repetitions. The constant stimuli method implementation is the "*ConstantStimuli*" class, derived from the "*PsyMethod*" class, without any addition. The QUEST method’s implementation that SALLO includes relies on a set of dedicated classes involving multiple programming languages. The QUEST algorithm comes from the "VisionEgg" Python 2.7 package (Straw, 2008). It can run in Unity thanks to the Unity extension "Python for Unity" 2.1.1 (Unity Technologies, 2019d). The session-specific instance of the QUEST algorithm runs in a separate Python thread, and the C# class "*pyQuest*" works as a Unity-Python interface: it queries the Python thread and translates the obtained values from Python to C#. We used the Python code for the QUEST algorithm and implemented the Python-Unity C# interface because we could not find any C# open-source implementation.

#### Positioning

The features described previously are minimal for a generic task to work. However, since the SALLO suite focuses on spatial orientation, it must also consider the spatial properties in the experimental design. SALLO includes object-related and observer-related spatial properties that let the experimenters track and guide the position of the entities in the virtual environment without additional code, with specific features tailored to the entities’ types.

##### Observer-related spatial properties

SALLO includes tools to track, guide, and react to the observer's movements. The tool to track the observer's movements is the "*PositionWatcher*" Component. It keeps track of the observer's orientation and signals if the observer exits from a given range. Moreover, "*PositionWatcher*" partners with another component, "*PitchController*", to provide acoustic pitch-based feedback if the experimental design requires the participant to have a specific orientation. Instead, the visual feedback for participant orientation guidance is a GameObject pointer in the form of a dark grey rectangle, slightly larger than the field of view, with a red dot in the middle. Every "Task X" GameObject contains a GameObject pointer and has the "*PositionWatcher*" and the "*PitchController*" Components attached (Fig. 3B).

##### Object-related spatial properties

SALLO offers several tools for GameObjects placement in the horizontal plane. Those GameObjects can be stimuli, "Task X" GameObjects, or other GameObjects used as reference frames. To cope with these three GameObject types' different requirements, SALLO treats them hierarchically according to their level of aggregation, that is, the amount of different GameObject types (i.e., stimuli, tasks, reference frames) the GameObject of interest can contain. The simplest GameObject is the stimulus, which should not contain any other GameObject type. The "Task X" GameObject follows since it can contain several stimuli. The reference frames close the hierarchy since they can contain a set of tasks or even a set of other reference frames. SALLO includes three components, each dedicated to specific levels of such hierarchy.

The first component is the class "*CylindricalCoordinates*"; it defines a GameObject position in terms of cylindrical coordinates (radius, angle, and elevation) instead of Cartesian coordinates (length, width, and elevation), the coordinate system Unity uses by default. "*CylindricalCoordinates"* encodes spatial orientation directly as an angle. Moreover, it contains a method to compute the stimuli radius in the virtual space according to the desired stimuli's visual angle. Since every virtual item can benefit from using cylindrical coordinates instead of cartesian ones, “CylindricalCoordinates” is useful at every aggregation level.

The second component is the class "*ArrayPlacer*"; it handles the spatial relationships among the items of a GameObject array, such as the angular distance among the array items or a common offset. With this tool, developers can control the relative position of, for example, the stimuli within a “Task X” GameObject or the multiple reference frames that the “Task X” GameObject can have.

The third component is the class "*Houser*"; it simplifies changing the GameObjects’ Parent GameObject among a set of available ones. It is useful in experiments with multiple reference frames to switch among them easily.

**Fig. 4:**
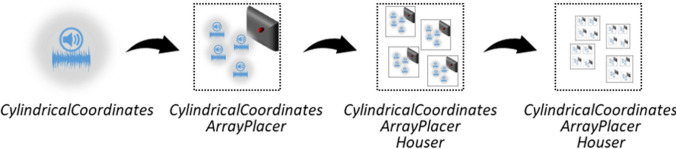
Hierarchical subdivision of SALLO GameObjects according to their level of aggregation and components implementing the stimuli-related spatial properties dedicated to each hierarchy level. The stimuli GameObjects require the *"CylindricalCoordinates"* component only; the "Task X" GameObjects require the previous component and, in addition, the *"ArrayPlacer"* component; The GameObjects used as reference frames require the previous components, and in addition, the *"Houser"* component

Figure 4 schematically highlights the positioning system's hierarchical structure and the dedicated tools.

### The SALLO front-end

The current section shows how to program an experiment with SALLO effectively and what it looks like. Notice that SALLO is a suite of tools that help design psychophysical experiments, not a standalone tool to run them; therefore, the final interface depends on the experiment-running tool used. SALLO uses an event-related paradigm, therefore it is virtually compatible with every experiment-running tool for Unity that employs events. However, SALLO was developed using the UXF as an experiment-running tool; therefore, this section will show how to use SALLO with UXF.

**Fig. 5:**
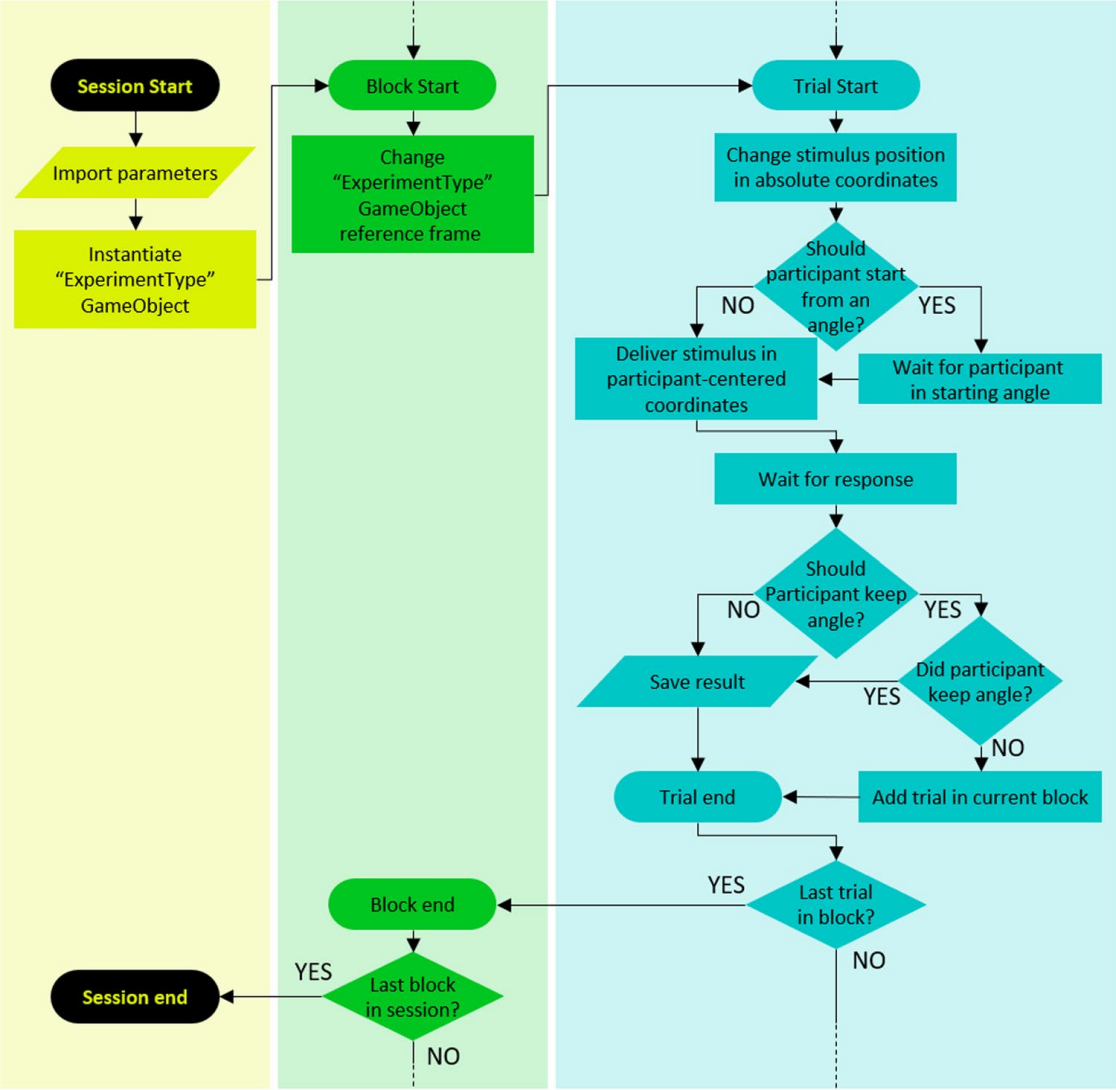
Flowchart outlining the events that compose an experiment using SALLO and UXF. The UXF events trigger the SALLO-based stimuli delivery. The SALLO-based stimuli delivery does not strictly depend on UXF but can be triggered using any other Unity asset based on events

The experimental design is defined entirely in the UXF experiment settings file. There are specific settings that every SALLO experiment requires, and they rule the experimental task in use, the sensory channel stimulated, the stimuli's temporal and spatial properties, the psychophysical method in use, and so on. The comprehensive settings list is reported in Table 1, with the required data type and an explanation for each setting variable. After choosing the proper values for the experiment settings, the experiment session follows the UXF session structure, which divides the session into blocks and the block in trials. The SALLO suite shapes the trials and blocks, introducing critical steps related to the stimuli delivery. The complete flowchart of a SALLO-UXF experiment is reported in Fig. 5.

**Table 1:** List of the parameters used in SALLO. To design an experiment, populate the experiment settings JSON file with the desired values

Name	Type—description
experimentType	string**—**the task for the experimental session. it must be the name of a prefab with a task-derived component
sensoryChannel	string**—**the sensory domain for the stimulus/i. it must be one of the “sensoryChannel” enum’s values: ‘acoustic’, ‘visual’, ‘audiovisual’ or ‘proprioceptive’
n_blocks	int**—**the number of experimental blocks, and therefore the number of reference frames
angle_between_blocks	float**—**the angular distance between each pair of reference frames from different blocks
angle_between_references	float**—**the angular distance between two reference stimuli *used only if experimentType is ‘Bisection’*
fov_angle	float**—**the stimulus’ angular width
Jitter	bool**—**choose whether stimuli position should be jittered
Maxjitter	float**—**the maximum amount of jitter for the stimuli position
procedure	string**—**the psychometric procedure for the test stimuli orientation selection to date, only the ‘Constant Stimuli’ and the ‘QUEST’ procedures are accepted if absent, ‘Constant Stimuli’ procedure is used
testing_angles	list < float **>—**the list of angles for the test stimulus *used only if procedure is ‘Constant Stimuli’*
Range	float**—**the range of values for the test stimulus’ angle, starting from 0. the stimulus will then appear in the interval [-range/2, + range/2] *used only if procedure is ‘QUEST’*
Grain	float**—**the minimum difference between two test stimulus angles *used only if procedure is ‘QUEST’*
addNoise	bool**—**choose whether gaussian noise should be added to the QUEST algorithm’s most informative value the noise variance is hardcoded, with standard deviation equal to 5% of range *used only if procedure is ‘QUEST’*
testing_trials	list < int** >—**the number of repetitions for each test stimulus angle. if the procedure is ‘Constant Stimuli’, each list item corresponds to the number of repetitions for the angle at the same index in the list testing_angles. if the procedure is ‘QUEST’, only one value is needed, and that’s the number of trials
checkConvergence	bool**—**choose whether to stop before the end of trials if a convergence condition is reached convergence condition hardcoded in the pyQuest class *used only if procedure is ‘QUEST’*
time_on	float**—**the time in seconds a stimulus is presented
time_off	float**—**in a sequence of stimuli, the time in seconds between two stimuli presentations
time_ITI	float**—**the time between the end of a trial and the start of the next one

The SALLO package contains the source code to run a sample experiment based on SALLO and UXF. The files provided with the sample experiment are the packages used (SALLO and UXF), the experiment settings files, the additional scripts used to setup and control the experiment flow, all the additional Unity files used in the Unity scene, and the Unity scene itself. Such a set of files is the set needed to ensure the study's reproducibility. In Unity, it can be easily exported as a unitypackage file and shared together with the article.

## Experimental use-case

The next section aims to showcase how researchers in the field of psychophysics may benefit from SALLO and VR to investigate the interactions among the multiple cues that shape the sense of spatial orientation. To do so, we describe an experimental use case that employs audiovisual stimuli whose position spans the whole frontal hemifield and depends on the head orientation. Performing such an experiment with physical stimuli would require using a large screen, multiple ones, or a small one that can move, paired with one large speaker array or a small one that can move. Such an apparatus is hardly portable; in fact, it may even require a dedicated room (Lewald et al., 2009; Populin, 2008). As this section will show, using VR made the whole setup much more portable, simpler in terms of hardware, and likely cheaper. The code used to run this experimental use-case is in the unitypackage file provided with this article's supplementary materials for reproducibility.

### Background

The human brain represents information concerning spatial orientation in multiple ways, typically divided into egocentric and allocentric representations (Klatzky, 1998). Egocentric representations encode spatial information with respect to the observer's point of view, e.g., "the car is on my right". Allocentric representations encode spatial information regardless of the observer's point of view, e.g., "the car is between the bicycle and the bus stop". Despite the allocentric representations' ideal independence from the observer, it has been shown that bodily inputs such as vestibulo-proprioceptive cues and motor efferent copies are involved in their neural computation (Ferrè et al., 2021; Lackner & DiZio, 2005; Laurens & Angelaki, 2018; Roncesvalles et al., 2005; Winter & Taube, 2014); therefore, the body posture, an inherently egocentric cue, may affect the estimation of allocentric representations as well. At the same time, it is unclear if the interaction between spatial reasoning and body posture differs when the spatial information is perceived via different sensory channels, that is, via vision or hearing (Cui et al., 2010; Lewald et al., 2009). The present study aimed to address both these open questions, focusing specifically on one effect: the distortion in the sense of spatial orientation arising from the orientation of the head on the trunk (Garcia et al., 2017; Lackner, 1974; Lewald et al., 1998, 2000; Odegaard et al., 2015; Schicke et al., 2002). This effect was chosen because it is consistent, and it has been replicated with a multitude of psychophysical tasks, such as head-pointing, pointer alignment, verbal reports, and so on (Garcia et al., 2017; Lackner, 1974; Lewald et al., 1998, 2000; Odegaard et al., 2015; Schicke et al., 2002). One psychophysical task that has been used to expose this effect is the left–right discrimination, where a stimulus appears at a given angle with respect to the physical median plane of the head of the participants, and they respond if they perceive the stimulus to the right or the left of their nose using a two-alternative forced-choice (2AFC) response pattern (Gescheider, 2013). This task has been used to investigate the head-on-trunk orientation-related distortion of the spatial orientation in the auditory domain, using both dichotic (Lewald et al., 2000) and free-field (Lackner, 1974) listening. Doing the task with the head turned with respect to the trunk has been shown to cause a shift in the psychometric curve's point of subjective equality (PSE), corresponding to the perceived head auditory median plane (HAMP) (Lackner, 1974; Lewald et al., 2000). The left–right discrimination task has never been employed in the visual domain to investigate the head-on-trunk orientation-related distortion of the perceived head visual median plane (HVMP). However, as there is evidence that the egocentric coordinates of hearing and vision can differ (Cui et al., 2010; Lewald et al., 2009), comparing how the head-on-trunk orientation affects the perceived median plane of the head (H*MP) depending on the perceptual modality involved can provide useful evidence to better understand the nature of such egocentric biases. In addition, the results obtained in the left–right discrimination task can be compared directly to those obtained in another task, the spatial bisection (Aggius-Vella et al., 2020; Amadeo et al., 2019; Gori et al., 2014; Rabini et al., 2019; Bertonati et al., 2023), which can be intended as its allocentric counterpart. The space bisection task consists of presenting three stimuli in sequence at monotonically increasing or decreasing angles and asking the participant which anchor stimulus, the rightmost or the leftmost, the second one was closer to. It can be intended as the allocentric counterpart of the left–right discrimination task because it differs from the latter only in the reference defining the left and right: oneself (egocentric reference) for the left–right discrimination, or the anchor stimuli (allocentric reference) for the space bisection. By testing participants on both the left–right discrimination and the space bisection tasks with different head-on-trunk orientations, using visual or acoustic stimuli, we investigated to what extent the head-on-trunk orientation effect depends on the spatial representation or perceptual modality involved. Specifically, in all the above mentioned conditions, we compared the difference in PSE obtained when the participants’ head was turned to the right or to the left. If the head-on-trunk orientation were perceptual modality-dependent, the differences in PSE obtained in the conditions with visual stimuli and those obtained with acoustic stimuli should differ. If the head-on-trunk orientation were spatial representation-dependent, the differences in PSE obtained in the left–right discrimination tasks and those obtained in the space bisection tasks should differ.

## Materials and methods

### Participants

In total, ten sighted individuals were involved in the study (five males, five females, age 34.7 ± 1.79 years old). The participants were enrolled from the local contacts of Genova. Informed consent was obtained from all of them. The study followed the tenets of the Declaration of Helsinki and was approved by the ethics committee of the local health service (Comitato Etico, ASL 3, Genova).

### Apparatus and setting

The study used Unity 2019 LTS on an Alienware 13 R3 laptop to run the experiment. An HTC Vive Pro head-mounted display (HMD) tracked the participant's head orientation and displacement in 3D and delivered the spatialized audio via integrated headphones. An XSENS MTw Awinda inertial measurement unit (IMU) (Paulich et al., 2018) tracked the participant's trunk orientation and linear acceleration in 3D. A backpack-like harness kept the IMU on the backbone at the shoulder level. The experiment was performed in a dimly lit and silent room. Participants sat on a chair for the whole experiment and were instructed to keep their torsos far from the chair's backrest during experimental blocks. They held the HTC VIVE pro controllers, one per hand.

The study used UXF and SALLO together to run the experiment in Unity. The virtual stimulus was SALLO's default: a light grey blurred sphere emitting intermittent white noise generated at runtime. The sphere was blurred utilizing a quad GameObject placed in front of the stimulus, whose shader implemented a Gaussian blur. The stimulus had the following physical properties: diameter of 30°, illuminance of 1.58 lx at the eye level, and volume of 70 dB_SPL_. A further audio-visual stimulus was used to guide the participants in their head orientation’s self-adjustment. The visual guide was a dark grey rectangle, slightly larger than the VR HMD’s field of view (FoV), with a circular pointer in the center. It was placed in the virtual space such that the pointer matched the desired angle and the rectangle matched the head orientation in 3D: the orientation was correct if the dark grey rectangle covered the whole FoV. The background rectangle’s illuminance was 1.05 lx at eye level. As the stimulus was displayed with the rectangle covering the FoV completely, the Weber contrast was computed as the contrast between the background rectangle illuminance and the stimulus illuminance, and it was 0.5. The acoustic guide was a metronome-like sound whose pitch varied according to the angular distance between the instantaneous head orientation and the desired one, peaking at 0° distance. The guidance sound was 6 dB_SPL_ quieter than the stimulus. The visual rendering was performed using Unity’s built-in rendering pipeline (Unity Technologies, 2019g). The "Resonance" audio plugin rendered the audio spatialization via a non-individualized head-related transfer function (HRTF). See Appendix A for a more comprehensive description of the virtual stimuli’s physical properties characterization.

**Fig. 6:**
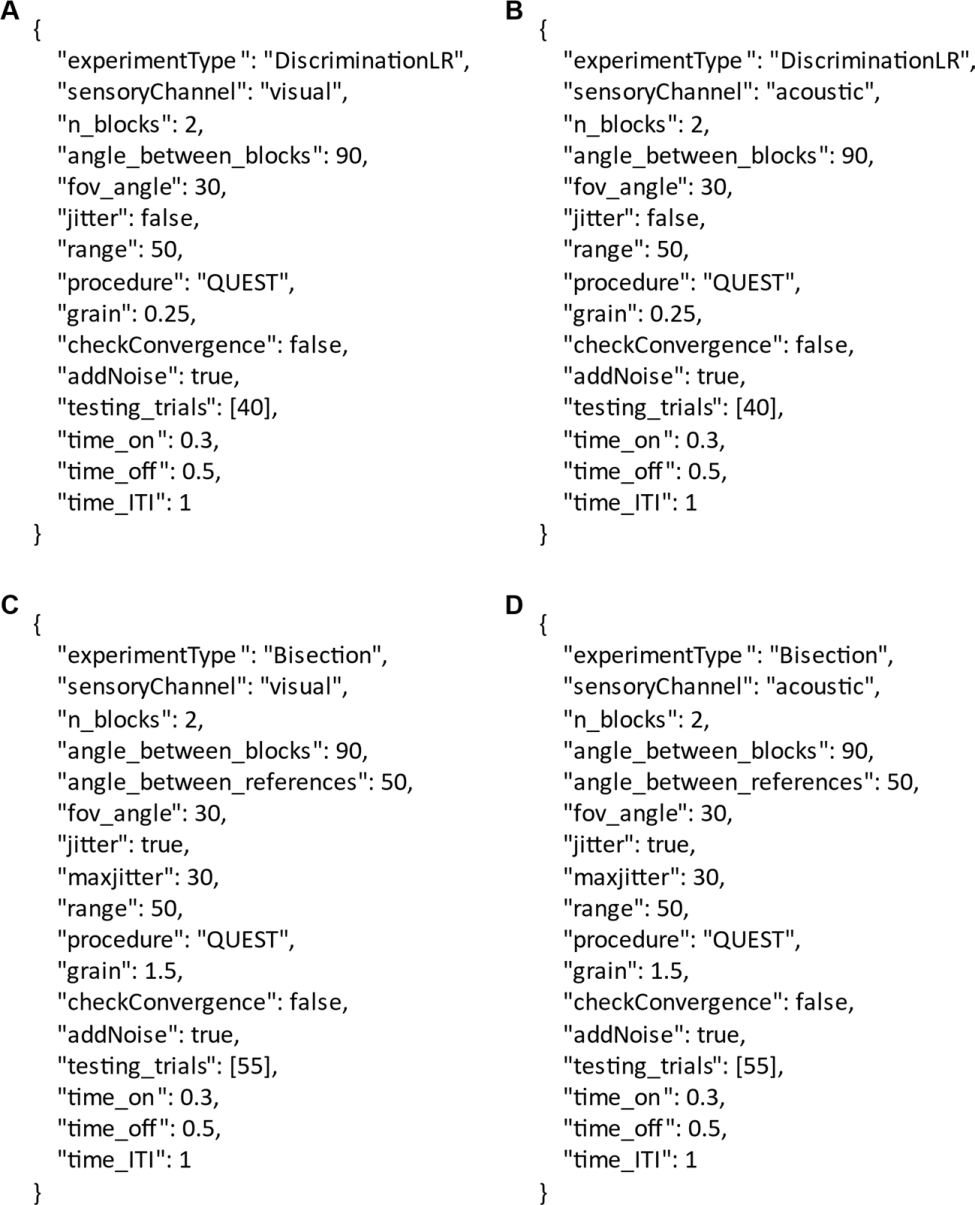
Content of the experiment settings file for the left–right discrimination task, visual (A) and acoustic (B), and for the space bisection task, visual (C) and acoustic (D)

The experimental design consisted of four tasks: left–right discrimination, visual and acoustic, and space bisection, visual and acoustic. They were implemented by parameterizing the UXF experiment settings file accordingly. The settings file content for the four tasks is reported in Fig. 6

### Experimental procedure

Apart from the stimuli modality and sequence and the question asked, the procedure used for the four SALLO-based experimental sessions was the same. Therefore, the common procedure is described first, and the task-specific aspects are described in separate paragraphs.

#### Common procedure

**Fig. 7:**
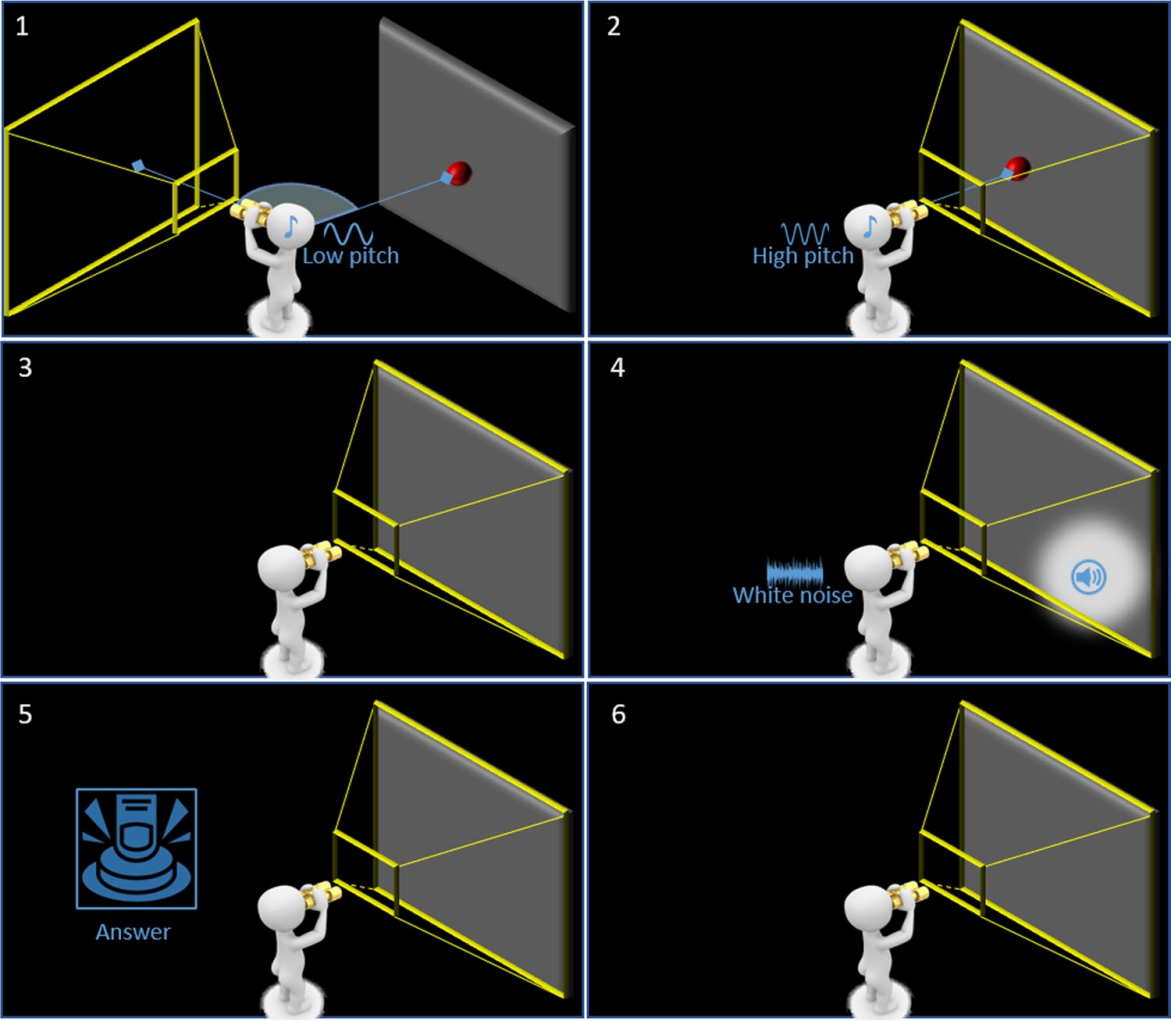
Stimuli delivery sequence common to all the tasks in the experimental use case. Even though the task in the experiment was only visual or only acoustic, to simplify the scheme comprehension, this figure reports the audio-visual cue. The participant uses the feedback to find the trigger orientation (1, 2). After a brief time interval of silence (3), the stimulus is shown for the requested time (4). The participant then provided the answer (5). After another short break of silence (6), the feedback guides the participant again toward the trigger position (1, 2)

The experimenter explained the task, verbally described the sounds at play, and prepared the participant. Then, the participant did some familiarization trials to understand the trial structure. Less than ten trials were enough in all cases. The trial was structured as follows. A guidance stimulus helped the participants orient their heads towards the reference angle for that experimental block, θ. θ was the same for the whole experimental block; therefore, participants were instructed to try and keep their heads at that orientation for the whole block duration. The guidance stimulus disappeared 1 to 3 s after the participant's head lay in an acceptability range of θ ± 3°; the stimuli delivery sequence started 1 s later. The stimuli were in head-centered coordinates, and their position depended upon the QUEST psychophysical adaptive procedure (Watson & Pelli, 1983), which indicated an orientation for the test stimulus close to the most informative orientation. Meanwhile, a parallel thread checked if the head orientation lay in the acceptability range θ ± 3° throughout the whole stimuli sequence delivery. The participant answered the task-related question by clicking the controller's trigger in the appropriate hand: left hand to answer left, right hand to answer right. No time constraints were placed upon the answer. After the participant answered if they kept the head in place during the stimuli sequence delivery, the trial parameters and results were saved, and the next trial started; otherwise, the current trial results were discarded, and the trial was added back to the current block's trials queue. The inter-trial interval time was 1 s. Figure 7 illustrates the common trial structure. The experimental session for each task consisted of two blocks: one with θ at -45° and one at + 45°. The blocks were counterbalanced among participants using partial randomization. A short break of no more than 5 min interspersed the experimental blocks and tasks. The execution order of the four tasks was counterbalanced using partial randomization. The whole SALLO-based experiment lasted around 60 min, breaks included.

#### Left–right discrimination task

The left–right discrimination task consists of estimating on which side a virtual sound stimulus is perceived with respect to the head's median plane. Specifically, participants were instructed to answer "right" if they heard the stimulus to the right of their nose or "left" vice-versa. The virtual stimulus lasted 300 ms. This value was chosen because it is a good trade-off between duration and spatial precision for the acoustic modality (Middlebrooks & Green, 1991) that ensures a balance between vision and hearing. Its position was chosen using the QUEST algorithm. The algorithm could sample from the range ± 30°, with a minimum step of 0.25°. Each block consisted of 40 trials. Of these trials, 4 were "catch trials" placed at -25° and at + 25° to ensure the task was performed correctly. The QUEST parameters were chosen based on results from pilot participants. The whole session lasted, on average, 10 min, with familiarization and breaks included.

#### Space bisection task

The space bisection task estimates the spatial relationship among three virtual stimuli appearing sequentially. Each stimulus lies at a monotonically increasing or decreasing angle from the previous one. The anchor stimuli are the first and last at the extreme positions. The second stimulus, lying between the anchors, is the test one. Participants were instructed to answer "right" if they perceived the test stimulus closer to the right-most anchor or "left" vice-versa. The virtual sounds lasted 300 ms, separated by 500 ms inter-stimulus intervals. The stimuli duration was chosen for consistency with the left–right discrimination task. The anchor stimuli were placed at 50° from each other, the same value used in previous studies (Aggius-Vella et al., 2020; Amadeo et al., 2019; Gori et al., 2014; Rabini et al., 2019), and could appear in the range ± 30° with respect to the anchors' midpoint to make sure the three stimuli could all appear in the same hemispace and therefore counterbalance differences in left and right space perception (Jewell & McCourt, 2000). The test stimulus position was chosen using the QUEST algorithm. The algorithm could sample the value in the range ± 25° from the anchors' midpoint, with a minimum step of 1.5°. Each block consisted of 55 trials. Of these trials, 4 were "catch trials" placed at -20° and + 20° to ensure the task was feasible and correctly performed. The QUEST parameters were chosen based on results from pilot participants. The whole session lasted, on average, 20 min, with familiarization and breaks included.

### Data analysis

As said above, the study aimed to investigate how the head-on-trunk orientation affects the PSEs of spatial tasks that use similar stimuli to probe different spatial representations through different perceptual modalities. The PSEs were computed as the medians of the psychometric curves fitted on raw data for each task, perceptual modality, experimental block, and participant. The psychometric functions were fitted using a cumulative Gaussian (Kingdom & Prins, 2016) with guess and lapse rate as fixed parameters constrained to the [0,0.1] interval and PSE and JND as free parameters. The bootstrap method was used to estimate the PSEs confidence intervals (Kingdom & Prins, 2016). The study compared the differences between the PSE obtained with the head turned to the right and the PSEs obtained with the head turned to the left (∆PSE) in a given condition. The ∆PSE distributions from different perceptual modalities and tasks were tested for normality with the Shapiro–Wilk test. Since the normality tests on the ∆PSE samples did not reach significance, the ∆PSE comparisons were conducted using the repeated measures ANOVA test, accompanied by the partial eta squared (η_p_^2^) as an estimate of standardized effect size. The post hoc analyses, instead, used the paired *t* test (t), accompanied by the Cohen’s *d* (*d*) as an estimate of standardized effect size. The psychometric curve fitting was performed in MATLAB r2020a (MATLAB, 2020) using a custom MATLAB function. The statistical analysis was performed in R (R Core Team, 2020).

## Results

The study analyzed the ∆PSEs, that is, the difference between the PSEs obtained with the head turned 45° rightward (PSE_+45_) and those obtained with the head turned 45° leftward (PSE_-45_). Table 2 shows the individual PSE_+45_ and PSE_-45_ estimates and their standard error for each task and perceptual modality. Positive PSE_+45_ and PSE_-45_ values indicate rightward shifts with respect to the real median plane of the head; negative values indicate leftward shifts. ∆PSE values larger than zero indicate that the PSEs shifted away from each other. ∆PSE values smaller than zero indicate that the PSE shifted toward each other.

**Table 2:** Individual PSE estimates (M) and their corresponding standard errors (SE) for each task, sense, and head-on-trunk orientation. Positive values indicate rightwise shifts with respect to the real median plane of the head; negative values indicate leftwise shifts. The values are in degrees (°)

[°]	ID	LEFT–RIGHT DISCRIMINATION				SPACE BISECTION			
		pse + 45		pse-45		pse + 45		Pse-45	
		M	SE	M	SE	M	SE	M	SE
VISUAL	s01	3.28	-0.39	2.12	-0.22	-15.72	2.45	-4.64	1.97
	s02	-0.56	-0.88	-2.04	-0.94	-3.48	-0.02	2.00	0.14
	s03	-0.56	-1.02	-0.12	-0.84	0.16	-0.15	0.60	-0.36
	s04	-0.48	-1.17	-0.32	-0.50	0.64	-0.02	-2.56	-0.26
	s05	2.00	-0.76	0.48	-0.57	0.92	0.02	-0.12	0.22
	s06	1.36	0.19	1.44	-0.52	2.52	0.01	3.36	0.83
	s07	-1.40	-0.27	0.00	-1.45	-1.96	0.50	0.00	0.13
	s08	1.00	-0.28	-1.36	-0.24	4.08	0.13	1.40	0.10
	s09	0.80	-0.60	-0.84	-0.99	-0.24	0.16	1.40	0.19
	s10	3.04	-0.42	-0.08	-0.34	-0.88	0.45	-0.88	0.27
ACOUSTIC	s01	2.64	0.05	0.08	0.38	1.04	1.37	1.92	0.65
	s02	-0.92	-0.41	-2.44	-0.67	-1.73	1.00	-2.92	0.52
	s03	-2.64	0.10	-2.16	-0.01	-1.68	0.40	-0.04	-0.12
	s04	1.92	-0.12	0.92	0.03	1.08	0.32	2.92	0.46
	s05	0.24	0.11	1.64	-0.62	0.68	0.13	4.48	0.40
	s06	-1.88	-0.39	-0.76	-0.14	5.12	1.78	8.24	0.38
	s07	-3.96	-0.17	-2.76	0.51	0.84	1.44	2.40	0.45
	s08	5.08	-0.14	3.64	0.81	1.68	0.84	4.34	0.56
	s09	-2.28	0.24	-3.20	0.01	-4.28	1.11	-2.52	0.15
	s10	1.20	0.01	-3.04	0.40	1.56	1.15	3.36	1.58

**Fig. 8:**
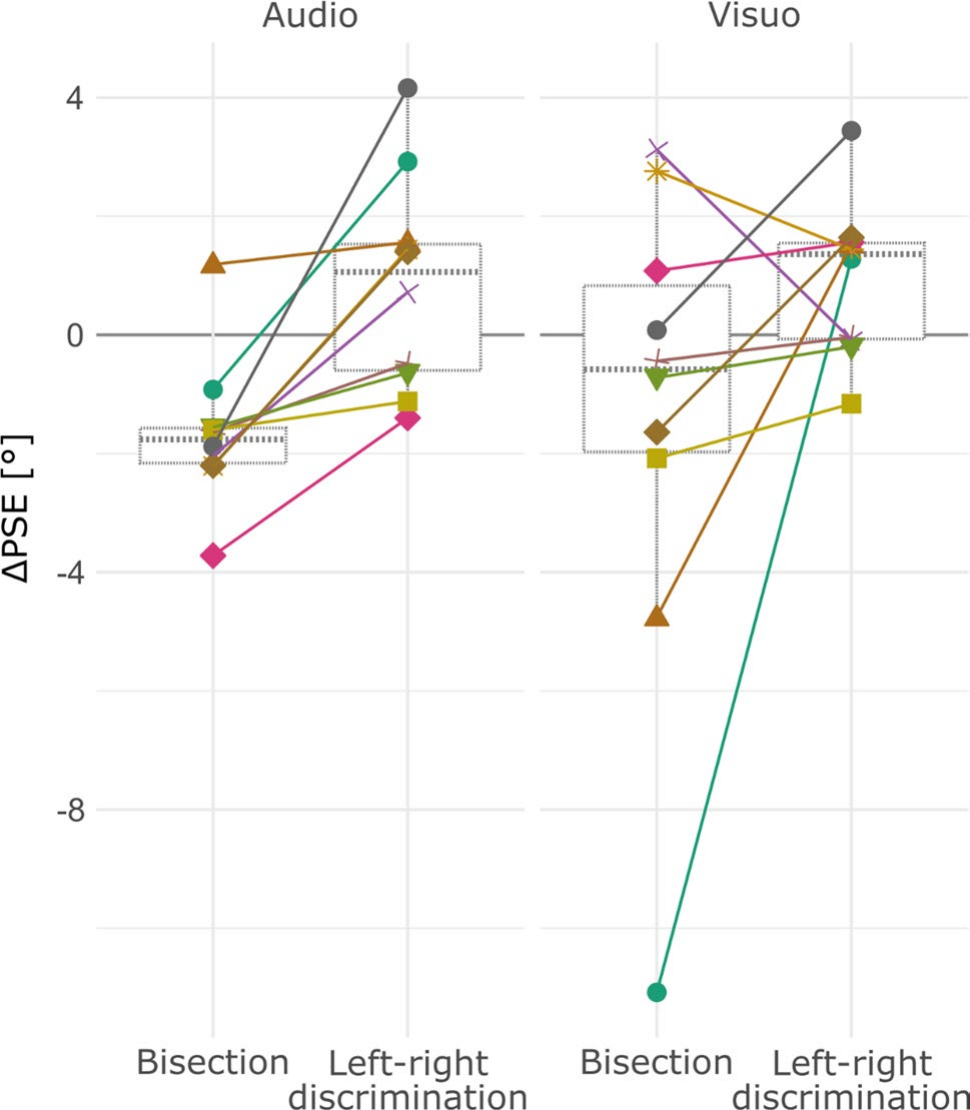
Boxplots and individual data points for the ∆PSEs (PSE_+45_—PSE_-45_) obtained in the left–right discrimination task and in the space bisection task in the acoustic and visual modalities. ∆PSE values larger than zero indicate that the PSEs shifted away from each other. ∆PSE values smaller than zero indicate that the PSE shifted toward each other. Concerning the individual data points, each shape-color pair indicates a different participant

The ∆PSEs (Fig. 8) were analyzed via a repeated measures ANOVA test with “sense” and “task” as within-subject effects. The repeated measures ANOVA test was significant for the main effect “task”, *F*(1,9) = 9.108, *η*_*p*_^*2*^ = 0.216, *p* = 0.015. It did not reach significance for the main effect “sense”, *F*(1,9) = 0.044, *η*_*p*_^*2*^ = 0.002, *p* = 0.838, nor for the interaction effect “task:sense”, *F*(1,9) = 0.021, *η*_*p*_^*2*^ = 0.001, *p* = 0.887.

The post-hoc analysis was conducted on the tasks’ “grand” ∆PSEs, computed as the average of the ∆PSEs that every individual obtained in each task's visual and acoustic modalities. The post-hoc analysis revealed that the ∆PSE distribution obtained in the left–right discrimination task (M = 0.898°, 95%CI [0.120,1.677]) was significantly larger (*t*(9) = 3.018, *d* = 0.95, *95%CI* [0.19, 1.79], *p* = 0.015) than the one obtained in the space bisection task (M = -1.51°, 95%CI [-0.010,-3.010]), that is, the PSEs shifted away from each other in the left–right discrimination and toward each other in the space bisection.

## Discussion

The present study aimed to showcase the SALLO suite utility for designing and developing psychophysical experiments focused on spatial orientation in VR. To do so, the study used SALLO, UXF, Unity, a commercial VR headset, and an inertial sensor to implement the visual and the acoustic versions of the left–right discrimination and of the space bisection tasks. The left–right discrimination task aimed to replicate the head-on-trunk rotation-related distortion of the egocentric space in both the acoustic and the visual domains; the space bisection task aimed to extend the literature by investigating if the head-on-trunk rotation could affect the allocentric reasoning as well.

**Fig. 9:**
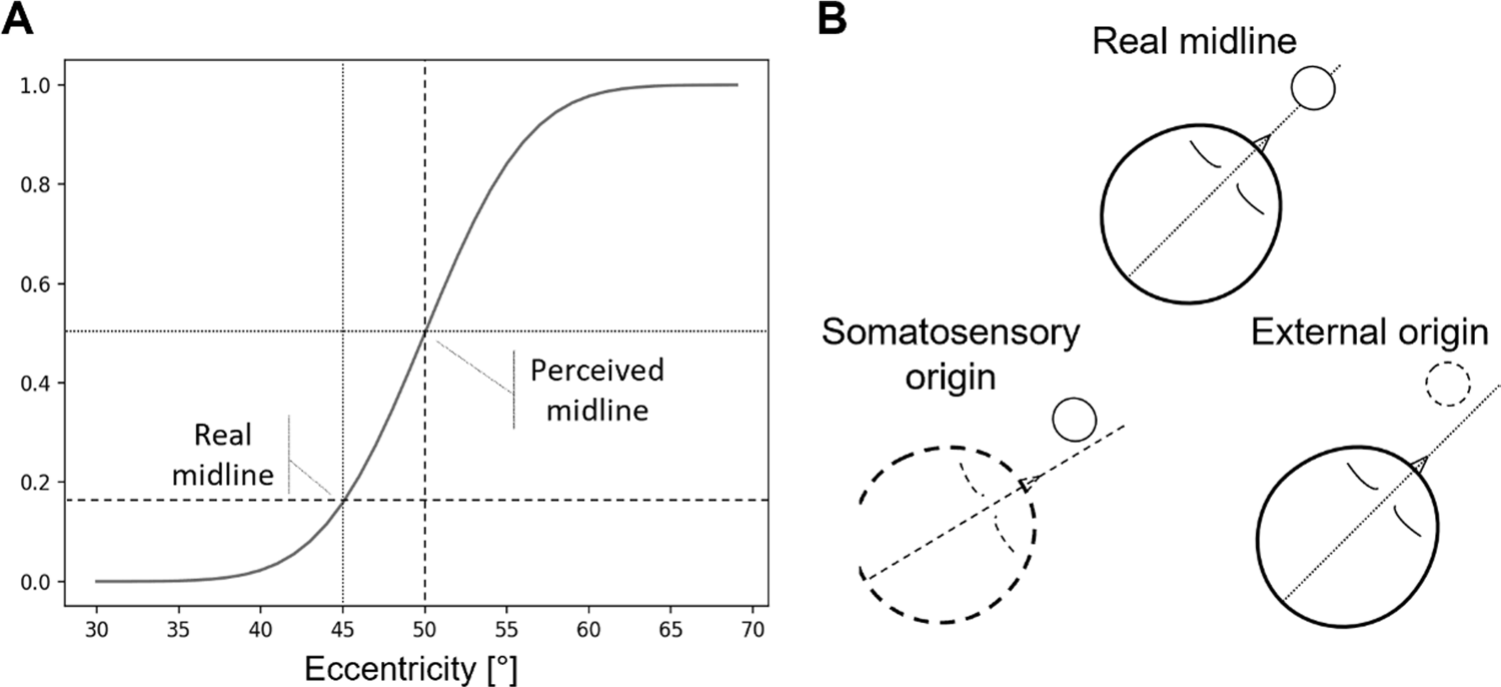
Graphical description of the effect found in the left–right discrimination task. (**A**) When the PSE is more eccentric than the physical midline, the observers tend to perceive a stimulus presented on the physical midline as less eccentric than it is, that is, they underestimate the stimulus eccentricity with respect to the perceived midline. (**B**) Such underestimation may have a somatosensory origin, that is, it may arise from the overestimation of the head rotation, it may have an external origin, that is, it may arise from the compression of the external space, or it may have both origins

The study found a significant positive ∆PSE in the left–right discrimination task, thus replicating the head-on-trunk rotation-related distortion of the egocentric space previously reported (Lackner, 1974; Lackner & DiZio, 2005; Lewald, 2002; Lewald et al., 1998, 2000), without significant differences between perceptual modalities, suggesting that the process causing the egocentric space distortion takes place similarly for both the acoustic and the visual modalities. The effect replication showed SALLO's reliability for conducting studies about the influence of low-level bodily cues on spatial encoding. That being said, in the left–right discrimination, the effect direction is not so informative per se since the literature has reported distortion effects with different directions depending on the specificities of the paradigm in use, like the acoustic stimulation method (free-field vs. dichotic listening (Lackner, 1974)) or the kinematics of the head rotations (Lackner, 1974; Lackner & DiZio, 2005; Lewald et al., 2000). Such variability in the literature may depend on the different parameterizations of the same task affecting the bodily and the spatial cues differently. Unfortunately, the left–right discrimination alone does not reveal if the effect found underlies a distortion of the body or the space perception. Taking as an example the positive ∆PSE observed here, it can be explained by the underestimation of the stimuli eccentricity with respect to the head, the overestimation of the perceived head-on-trunk orientation with respect to the stimuli, or both (Fig. 9). Further dedicated investigations are required to address how bodily and spatial cues contribute to the outcome of the left–right discrimination task.

The study found a significant negative ∆PSE in the space bisection task. This result confirmed our hypothesis that the allocentric representation is not “pure”, that is, completely observer-independent (Filimon, 2015), but rather that it can be affected by the body posture, as the neuroanatomical and neurofunctional evidence from the literature suggested (Ferrè et al., 2021; Lackner & DiZio, 2005; Laurens & Angelaki, 2018; Roncesvalles et al., 2005; Winter & Taube, 2014). In particular, the presence of an effect in the space bisection task supports the idea that the effect has an external origin (external space compression), as a somatosensory origin (head-on-trunk rotation overestimation) would have affected the external stimuli equally, and therefore would not have affected their perceived relative distance. Moreover, the effect's negative direction in the space bisection is consistent with the positive direction in the left–right discrimination, as it can be attributed to the compression of the distance between the anchors driven by the peripheral anchor’s eccentricity underestimation. While we could not find studies addressing similar effects concerning allocentric reasoning, the literature provides relevant evidence about the egocentric processing of peripheral stimuli. In those studies, it has been shown that the eccentricity of both acoustic and visual stimuli can be underestimated as long as the eccentricity is computed with respect to the body midline (Becker & Saglam, 2001; Esposito et al., 2023; Occhigrossi et al., 2021). In light of such literature, we propose that the effect we found in the space bisection underlay a two-stage process where the external stimuli were encoded in an external egocentric space first, and then the allocentric judgments were performed based on the stimuli positions in the external egocentric coordinates. This interpretation is in line with the research of Aggius-Vella, who found that the performance difference in the acoustic space bisection task changes if the stimuli are presented to the participants' front or to their back (Aggius-Vella et al., 2018, 2020), suggesting that the allocentric space representation is in fact anisotropic in egocentric coordinates.

In conclusion, the study replicated the previous literature about the effect of head-on-trunk orientation on egocentric reasoning and showed that allocentric reasoning is also affected. Moreover, the effect directions suggested that the allocentric estimates may rely on an intermediate processing stage that encodes the objects’ position in space in egocentric coordinates. Other dedicated experiments are required to test the latter hypothesis, and in this regard, SALLO provides an optimal framework since it makes it easy for experimenters to change stimuli and tasks independently, thereby simplifying the decoupling of the somatosensory, external egocentric, and allocentric processing of low-level cues.

## Conclusion

The study introduced SALLO, a suite of tools for the (multi-modal) psychophysical assessment of the effect of bodily cues on spatial orientation in auditory, visual, and audio-visual VR. It guides and simplifies the experimental paradigm design, providing utilities that, altogether, take care of all the necessary steps: stimuli choice, stimuli delivery, answer collection, and spatial properties. SALLO guides the experimenters in their VR-based psychophysics experimental design and saves them from implementing every step from scratch with general-purpose, dispersive tools. An experimental use case demonstrated the reliability of SALLO-based experiments in probing the contribution of low-level bodily cues to spatial orientation and the utility of SALLO in this regard. It did so by replicating an effect well established in the literature: the distortion of the egocentric space due to the head-on-trunk orientation (Lackner, 1974; Lackner & DiZio, 2005; Lewald, 2002; Lewald et al., 2000). The experimental use case also contributed to the literature, showing that rotating the head affects the allocentric space. These results could be obtained by performing different tasks with the same stimuli and setting and just minimal differences in the software parameterization. By simplifying psychophysical testing in VR, SALLO proved itself a useful asset to rapidly prototype and run low-cost experiments requiring complex and expensive hardware. For this reason, it has the power to speed up the research about the contribution of low-level cues on spatial orientation. SALLO is an open-source project aiming to provide researchers with the tools needed to conduct their research more simply. To that aim, we plan to improve SALLO's core and extend the set of stimuli, tasks, and psychophysical methods. Hopefully, this will happen according to the needs of the future community of SALLO users and with their help.

## Figures and Tables

**Fig. 10:**
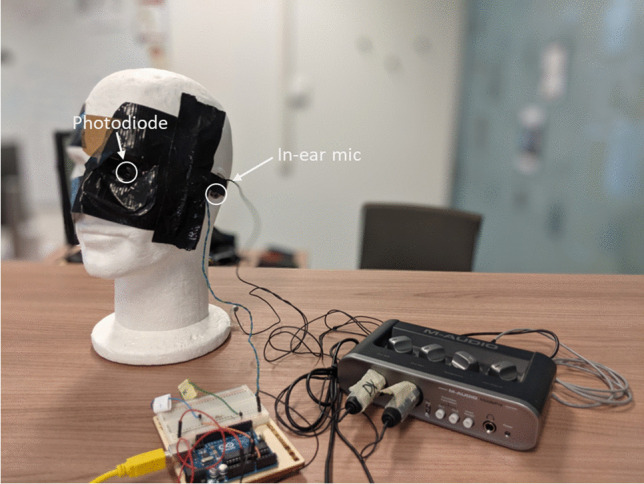
Apparatus used for the characterization of the VR stimulus’ audio-visual properties

## Data Availability

The raw and processed data sets and the statistical analysis code are openly available in the “Zenodo” repository at the URL https://doi.org/10.5281/zenodo.7152647. The experiment was not pre-registered.

## Appendix A: characterization of the stimulus’ audio-visual properties

To ensure the reproducibility of psychophysical studies, it is important to characterize the physical properties of the stimuli delivered. Typically, the physical properties are measured at the source. This is impossible for extended reality (XR) as the source is simulated, and, to date, no standard procedures for such measurements have been established yet. In this study, we measured the audio-visual properties of the stimuli at the receptor level rather than at the source. We did so by following a procedure similar to one of those that Murray, Patel, and Wiedenmann suggested in their work on how to calibrate the HMDs’ luminance (Murray et al., 2022). They made a pinhole camera out of a dummy head, embedding the necessary sensors in it. We readapted their approach for the measurement of both acoustic and visual characteristics. The characteristics we measured were the Weber illuminance contrast and the sound intensity. The following section describes the procedure used to measure them.

### Apparatus

The sound intensity measurement used a pair of SP-TFB-2 low noise in-ear binaural microphones, a M-AUDIO Mobile Pre mk2 USB audio interface, and the measurements were visualized in a DELL Latitude 3380 laptop running the software Audacity. The illuminance measurement used a Vishay BPW34 PIN photodiode, a 1 MΩ resistor, an Arduino UNO microcontroller, and the readings were visualized in the same DELL Latitude 3380 laptop running the Arduino IDE (Fig. 10).

The stimuli under measurement were presented using the same apparatus used in the experiment.

### Procedure

#### Sound intensity

The in-ear microphones were placed on the dummy head’s ear using tape. Then, the HMD was placed on the dummy head with the earphones covering the microphones, the acoustic stimulus was delivered, and the measured sound intensity was visualized in the laptop running Audacity.

#### Weber illuminance contrast

The photodiode was embedded in the dummy head’s left eye. The surface surrounding the “sensor eye” was covered in black tape to reduce stray light. Then, the HMD was placed on the dummy head, the visual stimulus was delivered, and the measured illuminance (lx) was visualized in the Arduino IDE serial port console. The illuminance was measured for the background and stimulus colors, viewed at full screen. The Weber contrast was computed using the following formula: $WC = \frac{{I}_{s}-{I}_{b}}{{I}_{b}}$, where I_s_ is the stimulus-related illuminance and I_b_ is the background-related illuminance.

## Code availability

The code used to run the experimental use-case is openly available in the “Zenodo” repository at the URL https://doi.org/10.5281/zenodo.8060398. SALLO is openly available and maintained in the git-hub repository at the URL https://github.com/DavideSpot/SALLO.git
